# Cancer Cell Membrane Biomimetic Mesoporous Nanozyme System with Efficient ROS Generation for Antitumor Chemoresistance

**DOI:** 10.1155/2022/5089857

**Published:** 2022-10-07

**Authors:** Wenxue Tang, Xiang Li, Meng Lyu, Qinqin Huang

**Affiliations:** ^1^The Research and Application Center of Precision Medicine, The Second Affiliated Hospital, Zhengzhou University, Zhengzhou, China; ^2^Department of Central Laboratory and Precision Medicine Center, Department of Nephrology, The Affiliated Huai'an Hospital of Xuzhou Medical University and Huai'an Second People's Hospital, Huai'an 223001, China; ^3^Department of Gastrointestinal Surgery, Shenzhen People's Hospital (The Second Clinical Medical College, Jinan University, the First Affiliated Hospital, Southern University of Science and Technology), Shenzhen, Guangdong 518020, China

## Abstract

Single-atom nanozymes (SAZs) with reaction specificity and optimized catalytic properties have great application prospects in tumor therapy. But the complex tumor microenvironment (low content of H_2_O_2_) limits its therapeutic effect. In this study, we developed a bionic mesoporous Fe SAZs/DDP nanosystem (CSD) for enhanced nanocatalytic therapy (NCT)/chemotherapy by simultaneously encapsulating the chemotherapeutic drugs cisplatin (DDP) and Fe SAZs with high peroxidase (POD) activity into the cancer cell membrane. CSD could evade immune recognition and actively targets tumor sites, and DDP upregulates endogenous H_2_O_2_ levels by activating nicotinamide adenine dinucleotide phosphate (NADPH) oxidase, thereby enhancing SAZs-mediated hydroxyl radical (·OH) production, which subsequently leads to mitochondrial damage and intolerance to chemotherapy drug. We used the HGC27/DDP cell line for in vitro and in vivo experiments. The results showed that CSD achieved good therapeutic benefits, without any side effects such as inflammatory reaction. This system can induce multiple antitumor effect with H_2_O_2_ self-supply, mitochondrial damage, and ATP downregulation and eventually lead to chemosensitization.

## 1. Introduction

With the development of anticancer drugs, the quality of life of cancer patients has been gradually improved, but the emergence of multidrug resistance (MDR) has brought difficulties to chemotherapy, which is also the main reason for the failure of chemotherapy [1–3]. MDR is characterized by the cross-resistance of cancer cells to a variety of anticancer drugs with different structures and mechanisms of action. The mechanisms of MDR include overexpression of drug efflux proteins, changes in apoptosis signaling, and activation of DNA repair mechanisms [4]. Methods to deal with MDR include the combination of chemotherapeutic drugs with efflux protein inhibitors, proapoptotic drugs, and tyrosine kinase inhibitors. However, simple combination chemotherapy is limited by the different pharmacokinetic properties of the drugs. Application of nanocarriers is a strategy to solve the above problems, such as liposomes, polymer micelles, mesoporous silica nanoparticles, gold nanoparticles, and cell membrane coating technology [5–9]. Loading drugs into traditional nanocarriers can achieve the same drug distribution in vivo, and nanoscale particles can also target tumor tissues through high permeability and retention effects [9, 10].

Nanozymes are a class of artificially simulated enzymes that have both the unique properties of nanomaterials and the catalytic efficiency and enzymatic reaction kinetics similar to natural enzymes [11]. Emerging single-atom nanozymes (SAZs), integrating single-atom technology and inherent enzyme-like active sites, elevate nanozyme technology to the atomic level and provide new opportunities to break through its inherent limitations. In SAZs, atomically dispersed metal centers maximize atom utilization efficiency and active site density [12]. SAZs have an excellent catalytic activity 10-100 times higher than that of traditional nanozymes [13, 14]. Metal-based SAZs have great potential to regulate tumor microenvironment to generate ROS such as ·OH, which could disrupt mitochondrial activity within tumor cells, thereby downregulating ATP synthesis; thus, redox microenvironment homeostasis was disrupted, and the end result is that tumor cells were killed through apoptosis or necrosis. In this process, MDR effect was synchronously tackled [15]. However, the ·OH content catalyzed by SAZs with peroxidase (POD) activity is highly dependent on the H_2_O_2_ content [16]. Intracellular reducing agents such as glutathione (GSH) balance the H_2_O_2_ level, which conspicuously suppressed SAZ-mediated ·OH production [17]. Therefore, improving the intracellular H_2_O_2_ concentration could effectively improve the therapeutic effect of SAZs.

**Scheme 1:**
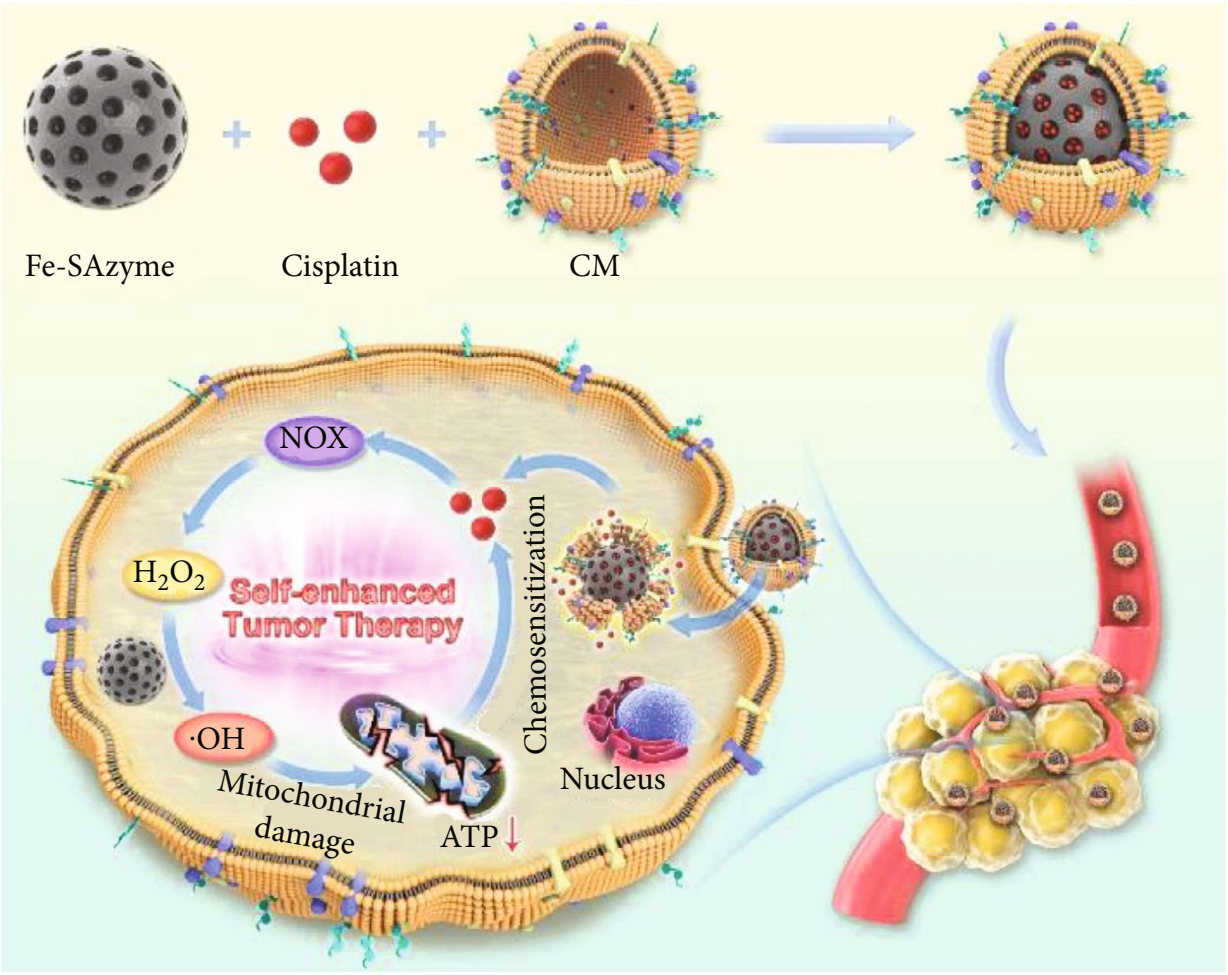
Cancer cell membrane biomimetic mesoporous nanozyme system with efficient ROS generation for antitumor chemoresistance.

In this study, a cancer cell membrane bionic mesoporous Fe SAZs/cisplatin cascade catalysis nanoplatform (CSD) was developed to weaken tumor resistance to DDP and remodel the TME for enhancing nanozyme-based NCT (Scheme 1). Tumor cell membrane biomimetic nanoparticles have good tumor homology targeting and biosafety and have been widely studied in recent years [18, 19]. CSD not only have homologous targeting at the cellular level, but at the animal level. As a typical platinum-based drug in clinical chemotherapy, cisplatin (DDP) can interact with the nuclear DNA of cancer cells to form Pt-DNA adducts that could cut off intracellular gene replication and transcription, thereby triggering many cellular responses, including cell cycle arrest, DNA inhibition replication, and transcriptional processes as well as apoptosis and necrosis [20–22]. It also activates nicotinamide adenine dinucleotide phosphate (NADPH) oxidase to generate superoxide anion (O_2_•^−^) [23, 24], which is subsequently dismutated by superoxide dismutase (SOD) to promote the production of H_2_O_2_. This motivates us to combine DDP with SAZs to satisfy both tumor catalytic therapy treatment and chemotherapy, as SAZs could destroy cell homeostasis to further weaken MDR and improve cisplatin resistance. In addition, CSD could successfully escape the interception of the liver and kidney, prolonging its circulation time in the body. Eventually, DDP upregulates H_2_O_2_ levels in the TME, and Fe SAZs can generate a large amount of ·OH, causing mitochondrial damage and downregulating ATP content, thereby improving the efficacy of DDP-mediated chemotherapy. This cascade reaction could achieve coadjutant NCT/chemotherapy and significantly inhibit tumor growth without physiological toxicity. This work achieves precise cancer treatment and enhanced efficacy of DDP through novel biomimetic nanotechnology, which has good clinical translation potential.

## 2. Results and Discussion

**Figure 1:**
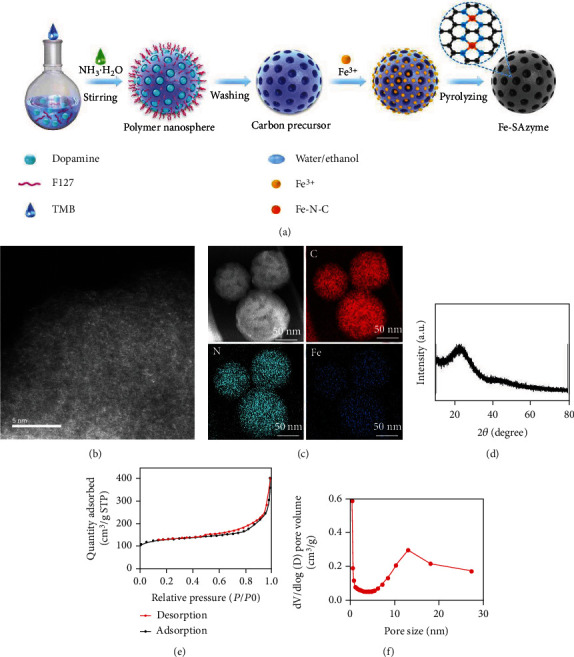
(a) Schematic illustration of Fe SAZ synthesis. (b) AC HAADF-STEM image of Fe SAZs. (c) HAADF-STEM image and corresponding EDS mapping of Fe SAZs. (d) XRD pattern of the Fe SAZs. (e) N_2_ absorption and desorption curves. (f) Pore size distributions for Fe SAZs.

SAZs have uniformly dispersed active sites and well-defined coordination structures, which can mimic the activities of various natural enzymes and can be used for biological detection and cancer treatment [25, 26]. The atomically dispersed Fe SAZs were obtained by firstly anchoring of iron ions on mesoporous carbon sphere precursors, followed by a pyrolysis process in an argon atmosphere, as shown in Figure 1(a). Firstly, mesoporous carbon sphere precursors were prepared by an organic-organic self-assembly approach that uses Pluronic F127 as soft template, ethanol/water mixture as solvent, and dopamine as carbon and nitrogen source, respectively. The precursor was then mixed with Fe^3+^ in water to anchor iron atom. Finally, the Fe SAZs were obtained by pyrolysis process at 600°C for 4 h. The isolated and high-density bright spots observed in aberration-corrected high-angle annular dark field scanning transmission electron microscopy (AC-HAADF-STEM) image (Figure 1(b)) implied the existence of single metal atoms. The uniform distribution of elements of C, N, and Fe in samples was observed in the EDS mappings (Figure 1(c)). Energy dispersive X-ray spectroscopy (EDX) (Figure S1) result shows that Fe SAZs are composed of two elements, namely, Fe and C. And XRD energy spectrum also showed no obvious diffraction peaks of Fe and its oxides, suggesting that Fe is distributed in atomic form (Figure 1(d)). The N_2_ adsorption-desorption measurements demonstrated a mesoporous size distribution of Fe SAZs (Figures 1(e) and 1(f)). According to the Barrett-Joyner-Halenda method, the specific surface area of prepared sample was calculated as 437 m^2^/g and has a fine pore structure. According to the results of ICP-AES, the iron content is about 1.1%.

**Figure 2:**
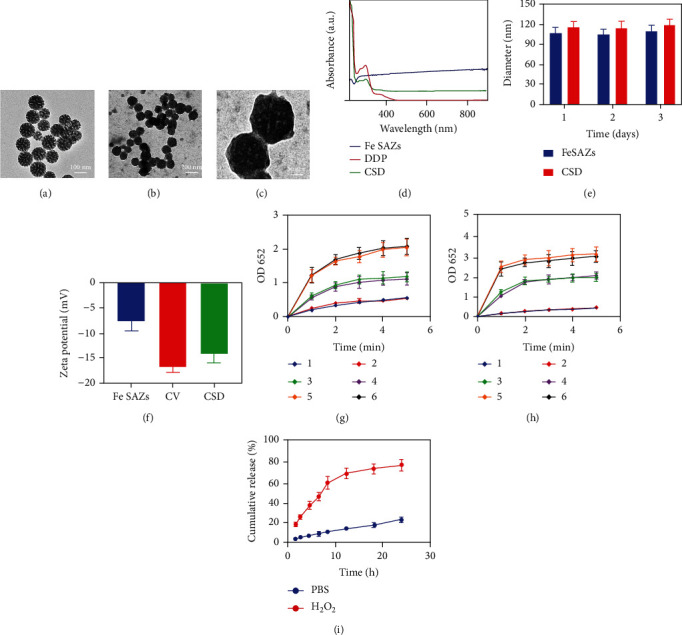
(a) TEM image of Fe SAZs. (b) TEM image of CSD. (c) High magnification TEM image of CSD. (d) Absorption spectra of Fe SAZs, DDP, and CSD in PBS. (e) Statistical graph of measured diameter size of Fe SAZs and CSD (*n* = 3). (f) Zeta potential values for Fe SAZs, CV, and CSD nanovesicles (*n* = 3). (g) Under different H_2_O_2_ conditions, the absorption peak at 652 nm in the chromogenic reaction of TMB involving Fe SAZs and CSD (*n* = 3; 1: Fe SAZs, 1 mM H_2_O_2_; 2: CSD, 1 mM H_2_O_2_; 3: Fe SAZs, 2 mM H_2_O_2_; 4: CSD, 2 mM H_2_O_2_; 5: Fe SAZs, 5 mM H_2_O_2_; and 6: CSD, 5 mM H_2_O_2_). (h) Under different pH conditions, the absorption peak at 652 nm in the chromogenic reaction of TMB involving Fe SAZs and CSD (*n* = 3; 1: Fe SAZs, pH = 7; 2: CSD, pH = 7; 3: Fe SAZs, pH = 6.5; 4: CSD, pH = 6.5; 5: Fe SAZs, pH = 5.5; and 6: CSD, pH = 5.5). (i) In vitro DDP release profile in the presence and absence of H_2_O_2_ (*n* = 3).

Cell membrane-coated biomimetic nanoparticles are mainly composed of a layer of cell membrane wrapping functional nanoparticles, thus forming a nanoparticle core-cell membrane-shell structure [27, 28]. In this study, we first prepared cancer cell membrane vesicles (CV) and then encapsulated Fe SAZs and DDP into CV to obtain a biomimetic hybrid material CSD. Transmission electron microscopy (TEM) image of as-prepared Fe SAZs is shown in Figure 2(a). And CSD showed a clear gray membrane coating on the outside of the Fe SAZs (Figures 2(b) and 2(c)). The drug loading efficiency of CSD was about 17.2% calculated by UV-Vis absorption spectroscopy (Figure 2(d)). The size of Fe SAZs and CSD was quantitatively calculated for three consecutive days (Figure 2(e)), and the results showed that the single-atom enzyme we prepared had good stability. The zeta potential of Fe SAZs was about -12.5 mV, and the zeta potential of CSD modified by cancer cell membrane was close to the level of CV (Figure 2(f)). Cancer cell membrane marker, Epcam, was detected using western blotting (Figure S2). This also indicates successful encapsulation of the cell membrane. The tumor microenvironment (TME) is different from the physiological environment of normal cells [14]. Due to the insufficient supply of nutrients and oxygen at the tumor site, glycolysis produces lactic acid, so the pH of the tumor site is usually lower than that of the normal adjacent tissues, showing a weak acid trend [29]. We carried out TMB (3,3′,5,5′-tetramethylbenzidine) chromogenic reaction experiments under different pH conditions or H_2_O_2_ concentration to explore the POD activities of CSD and Fe SAZs. The results showed that the POD activities of Fe SAZs and CSD enhanced with the increase of acidity and H_2_O_2_ (Figures 2(g) and 2(h)). DDP can disrupt redox homeostasis, upregulate NADPH oxidase activity (Figure S3), and stimulate more H_2_O_2_ production. And the coincubation of CSD and H_2_O_2_ will gradually release DDP, which is because the ROS generated by CSD could damage the cell membrane (Figure 2(i)).

**Figure 3:**
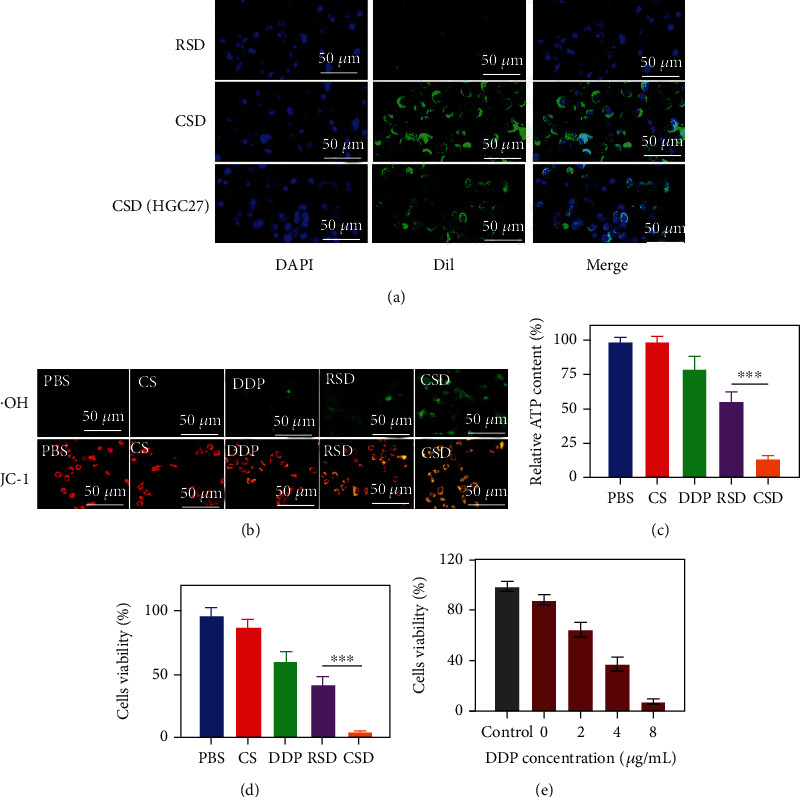
(a) Representative fluorescence images of HGC27/DDP cells after incubation with RSD or CSD for 2 h. Scale bars: 50 *μ*m. Cells were stained with DAPI (blue: cell nucleus; green: Dil). (b) Fluorescence image of hydroxyl radicals (·OH) and JC-1 in cells. Scale bars: 50 *μ*m. (c) ATP inhibition ability of different formulations (*n* = 3). (d) Cell viability of HGC27/DDP cells following the indicated treatments (*n* = 3). (e) Cell viability of HGC27/DDP cells following the CSD treatments under different DDP concentrations (*n* = 3). ^∗∗∗^*P* < 0.005.

**Figure 4:**
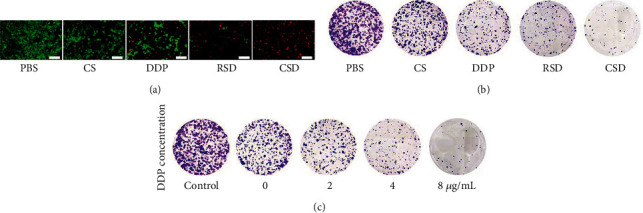
(a) Fluorescence images of HGC27/DDP cells stained with FDA (live cells, green fluorescence) and PI (dead cells, red fluorescence) after incubation with different formulations. Scale bars: 200 *μ*m. (b) Colony of HGC27/DDP cells treated with different formulations. (c) Colony of HGC27/DDP cells following the CSD treatments under different DDP concentrations.

Usually, the immune system recognizes and eliminates tumor cells in the TME [30]. However, in order to survive and reproduce, tumor cells can employ different strategies to suppress the body's immune system so that it could not properly recognize and kill tumor cells, thereby surviving all stages of the antitumor immune response [31–33]. The above-mentioned characteristic of tumor cells is called immune escape. Tumor cell membranes retain cell adhesion molecules on the surface of the source cell, including cadherins, selectins, integrins, and immunoglobulin superfamily. These receptors enable tumor cell membrane-coated nanoparticles to escape immune clearance and exhibit cognate targeting behaviors that greatly enhance their cancer-specific accumulation and retention [18, 34, 35]. We prepared erythrocyte membrane-coated Fe SAZs and DDP by a similar method, named RSD. Fe SAZs and DDP were coated with cancer cell membrane extracted from HGC27 cell line to form CSD (HGC27), which were used to function as control groups to verify the targeting ability of CSD. CSD, CSD (HGC 27), and RSD were labeled with Dil and coincubated with tumor cells (Figure 3(a)). The results showed that CSD is more easily phagocytosed by tumor cells than RSD as there are no receptor proteins on the erythrocyte membrane that recognize cancer cells. In addition, the number of CSD (HGC27) phagocytosed by HGC27/DDP was less than CSD, which may be due to the membrane protein change. In vitro cell uptake experiments were carried out with CSD, CSD (HGC 27), and RSD containing different concentrations of DDP, and the results also showed that CSD had the best HGC27/DDP cell-targeting ability (Figure S4). Next, we detected ·OH formation of different groups with the hydroxyphenyl fluorescein (HPF) fluorescent-staining experiment; the result showed that CSD-induced green fluorescence was the brightest (Figure 3(b)). Mitochondria, as the energy-producing structures of cells, are the main sites of cellular aerobic respiration. High concentrations of ·OH trigged by CSD group could damage mitochondria (Figure 3(c)), then the energy production channels of mitochondria are inhibited, and the content of ATP in cells is significantly reduced (Figure 3(d)). While RSD has a moderate tumor therapeutic effect, CSD with good homologous targeting ability induced the admirable killing efficiency. Initially, CSD could be recognized and preferentially phagocytosed by cancer cells, causing the release of DDP and Fe SAZs. Fe SAZs could catalyze the production of toxic ·OH from endogenous H_2_O_2_, and the subsequently generated ·OH in situ could disrupt mitochondrial activity, reducing ATP content. Simultaneously, DDP upregulate NADPH activity, which would induce tumor cells to produce more H_2_O_2_; this repeated cycle of treatment could continuously replenish H_2_O_2_ and maximize the effectiveness of DDP, thereby profoundly killing tumor cells. Cell viability in the CSD group was less than 15%, indicating that our complementary treatment was found to be effective for achieving enhanced NCT/chemotherapy. Similarly, we adjusted the loading of DDP in CSD, the concentrations of DDP were 0, 2, 4, and 8 *μ*g/mL (Figure 3(e)), respectively, and cell viability decreased with increasing DDP concentration without exhibiting drug resistance. Similarly, we detected the fluorescence images of HGC27/DDP cells stained with FDA and PI under different formulations; the results showed that CSD could maximumly eliminate cancer cells (Figure 4(a)). Colony formation assay also demonstrated consistent results (Figures 4(b) and 4(c)).

**Figure 5:**
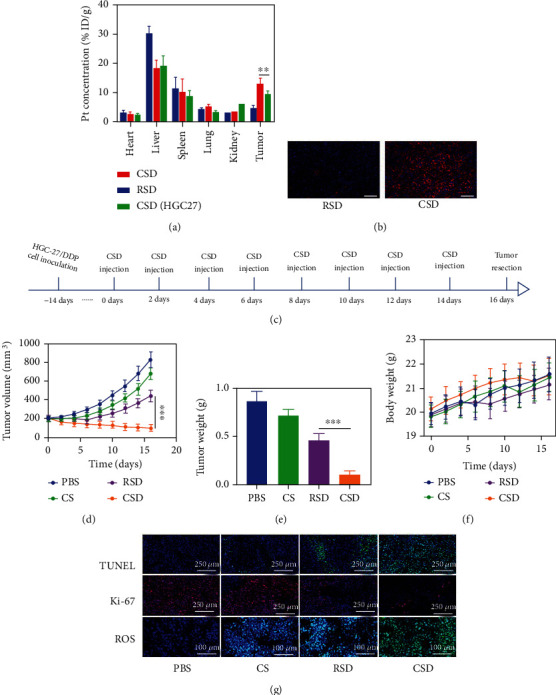
(a) Biodistribution profile of Pt element in main organs and tumor tissues (*n* = 5). (b) In vivo imaging of tumors treated with Dil-labeled RSD and CSD at 12 h postinjection by confocal laser scanning microscopy (blue: DAPI; red: Dil). Scale bars: 100 *μ*m. (c) The treatment schedule. (d) Relative changes of tumor volume in mice bearing HGC27/DDP tumors after indicated treatments (*n* = 5). (e) Tumor weight measured following the indicated treatments. (f) Representative digital photos of tumors collected from various groups (*n* = 3). (g) TUNEL, Ki-67, and ROS-stained tumor sections from the indicated treatment groups. ^∗∗∗^*P* < 0.005.

The good in vitro experimental results of CSD motivated us to continue to explore the tumor accumulation ability and therapeutic efficiency of CSD in vivo. First, a subcutaneous tumor model was established in Balb/c nude mice. We explored the in vivo pharmacokinetics and biodistribution of CSD and RSD. Due to the coating of the cell membrane, both CSD and RSD could significantly improve the circulation time in the body and reduce the accelerated blood clearance (ABC), thereby showing good long-term blood circulation. In vivo biodistribution experiment also showed that CSD and RSD achieved better immune evasion. Compared to RSD, only a small amount of CSD and CSD (HGC-27) accumulated in liver. However, RSD will not actively target tumor sites, and the constructed biomimetic nanoparticle CSD could better retain key proteins on the HGC27 cell membrane and escape immune clearance, and more importantly, CSD could specifically target drug-resistant tumor cells, resulting in better tumor accumulation (Figures 5(a) and 5(b)). In addition, ordinary cell membrane-encapsulated CSD (HGC27) have weak tumor targeting properties, which also reflects the homologous targeting ability of CSD. These results encouraged us to continue our in vivo antitumor experiments. The animals were randomly divided into four groups, each of which was subjected to one of the following treatments: (1) PBS, (2) CS, (3) RSD, and (4) CSD. When the tumor volume in these groups grew up to about 200 mm^3^, treatments were administered intravenously to each group. We measured tumor growth curves and mouse body weight every two days during antitumor period. As shown in Figure 5(f), the mice body weights did not exhibit excessive fluctuations during the treatment period and were not affected by the various treatments. This is very important. Although many nanomaterials have good killing effect on tumors, they also have corresponding physiological toxicity, which limits their long-term biological and clinical value [36]. The treatment schedule is shown in Figure 5(c). As shown in Figure 5(d), the tumor volume of the control group increased within two weeks, while the CS group containing only Fe SAZs showed almost no visible tumor suppressive effect, which is due to insufficient H_2_O_2_ content in the tumor physiological environment [37–39], Fe SAZ-mediated ·OH production was little. Notably, the RSD group achieved a moderate tumor suppression, and tumor volume in the CSD group was significantly suppressed during the treatment cycle. Cancer cell membrane-encapsulated CSD achieved immune escape and homologous targeting capabilities and accumulated specifically at tumor sites, greatly improving their cancer therapeutic effects. And Fe SAZ-mediated nanozyme-catalyzed therapy and DDP-mediated chemotherapy complement each other and improve the TME during the treatment, further increasing the corresponding effect of Fe SAZs/DDP. After the treatment, the tumors were weighed, and it was found that the average tumor mass in the RSD group was about 0.47 g, while that in the CSD group was only about 0.1 g, which was consistent with the tumor volume in mice (Figure 5(e)). Terminal deoxynucleotidyl transferase-mediated dUTP-biotin nick end labeling (TUNEL) and Ki-67 staining of tumor tissues from all groups are shown in Figure 5(g); the CSD group showed a large amount of tumor cell apoptosis and lower cell proliferation effect. ROS can destroy the active components of tumor cells, causing damage to their cell membranes, nucleic acids, proteins, etc., eventually leading to cell death [40, 41]. TME stimulated by DDP upregulates H_2_O_2_ levels, thereby promoting the ROS content catalyzed by CSD. CSD exhibited the highest ROS fluorescence production, indicating that our prepared material is an ideal redox destroyer.

**Figure 6:**
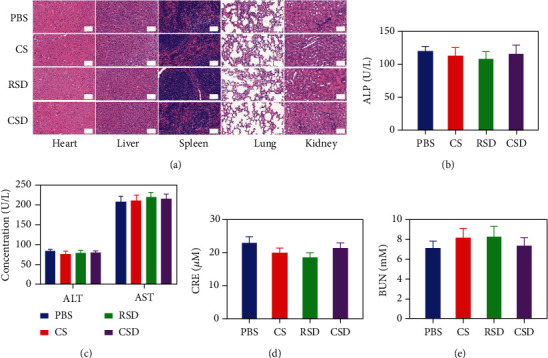
(a) Histopathologic examination of the tissues including the heart, liver, spleen, lung, and kidney from BALB/c nude mice after different treatments. Scale bars: 100 *μ*m. Blood biochemistry data including liver function markers ((b) ALP and (c) ALT) and AST and kidney function markers ((d) CRE and (e) BUN) from BALB/c nude mice after different treatments.

After the treatment, slices of the basic organs (heart, liver, spleen, lung, and kidney) of the control group and CSD group were taken for pathological toxicity analysis, as shown in Figure 6(a). There were no obvious physiological and morphological changes and inflammatory responses in both groups. And we conducted further blood biochemical analysis (Figures 6(b)–6(e)). All the indicators were normal. This result shows that the health of the mice was not affected after the treatment. The CSD we prepared not only has a good tumor inhibition rate but also exhibits far-reaching biosafety. The nanocarrier can maintain good biocompatibility of cell membranes, has relatively low cytotoxicity and immunogenicity, and would not cause significant side effects and rejection, reducing drug accumulation and toxic side effects in internal organs.

## 3. Conclusion

In conclusion, we have designed a cancer cell membrane biomimetic mesoporous single-atom nanozyme drug delivery system for self-enhancing catalytic therapy of drug-resistant tumors. CSD with cancer cell membrane modifications are more easily recognized and phagocytosed by tumor cells, subsequently release DDP and SAZs, and produce a domino-linked cell-killing effect. Firstly, DDP activated NADPH oxidative stress, thereby promoting more H_2_O_2_ production. Then, Fe SAZs catalyze the H_2_O_2_ to generate a large amount of ·OH, thereby killing tumor cell mitochondria and destroying ATP production, which also lead to a decrease in multidrug resistance of tumor cells and maximize the effect of DDP chemotherapy. This effective catalytic therapy provided a new insight for tackling multidrug-resistant tumors.

## Data Availability

All data reported in this study are available upon request by contact with the corresponding author. The detailed experimental procedures are included in the Supporting Information file.

## Supplementary Materials

Supplementary Materials Figure S1: EDX of Fe SAZs. Figure S2: cancer cell membrane marker, Epcam, was detected using western blotting. Figure S3: the activity analysis of NADPH oxidase (NOX) in HGC27/DDP cells after treated with 10 *μ*g/mL DDP for different time points. Figure S4: nanoparticle uptake by HGC27/DDP cells at different concentrations.

## References

[1] Tong Z., Gao Y., Yang H., Wang W., Mao Z. (2021). Nanomaterials for cascade promoted catalytic cancer therapy. *Viewpoints*.

[2] Ji B., Wei M., Yang B. (2022). Recent advances in nanomedicines for photodynamic therapy (PDT)-driven cancer immunotherapy. *Theranostics*.

[3] Wu W., Pu Y., Yao H., Lin H., Shi J. (2022). Microbiotic nanomedicine for tumor-specific chemotherapy-synergized innate/adaptive antitumor immunity. *Nano Today*.

[4] Qin X., Wu C., Niu D. (2021). Peroxisome inspired hybrid enzyme nanogels for chemodynamic and photodynamic therapy. *Nature Communications*.

[5] Ai Y., Hu Z. N., Liang X., Sun H. B., Xin H., Liang Q. (2022). Recent advances in nanozymes: from matters to bioapplications. *Advanced Functional Materials*.

[6] Sun K., Hu J., Meng X. (2021). Reinforcing the induction of immunogenic cell death via artificial engineered cascade bioreactor-enhanced chemo-immunotherapy for optimizing cancer immunotherapy. *Small*.

[7] Ding Y., Xu H., Xu C. (2020). A nanomedicine fabricated from gold nanoparticles-decorated metal–organic framework for cascade chemo/chemodynamic cancer therapy. *Advanced Science*.

[8] Wang X., Zhong X., Li J., Liu Z., Cheng L. (2021). Inorganic nanomaterials with rapid clearance for biomedical applications. *Chemical Society Reviews*.

[9] Wang X., Zhong X., Bai L. (2020). Ultrafine titanium monoxide (TiO1+x) nanorods for enhanced sonodynamic therapy. *Journal of the American Chemical Society*.

[10] Wang X., Zhong X., Cheng L. (2021). Titanium-based nanomaterials for cancer theranostics. *Coordination Chemistry Reviews*.

[11] Dai Y., Ding Y., Li L. (2021). Nanozymes for regulation of reactive oxygen species and disease therapy. *Chinese Chemical Letters*.

[12] Wu W., Huang L., Wang E., Dong S. (2020). Atomic engineering of single-atom nanozymes for enzyme-like catalysis. *Chemical Science*.

[13] Shen L., Ye D., Zhao H., Zhang J. (2021). Perspectives for single-atom nanozymes: advanced synthesis, functional mechanisms, and biomedical applications. *Analytical Chemistry*.

[14] Wang S. B., Chen Z. X., Gao F. (2020). Remodeling extracellular matrix based on functional covalent organic framework to enhance tumor photodynamic therapy. *Biomaterials*.

[15] Sharma A., Lee M.-G., Shi H. (2018). Overcoming drug resistance by targeting cancer bioenergetics with an activatable prodrug. *Chem*.

[16] Yu P., Li X., Cheng G. (2021). Hydrogen peroxide-generating nanomedicine for enhanced chemodynamic therapy. *Chinese Chemical Letters*.

[17] Tang Z., Zhang H., Liu Y. (2017). Antiferromagnetic pyrite as the tumor microenvironment-mediated nanoplatform for self-enhanced tumor imaging and therapy. *Advanced Materials*.

[18] Tian H., Luo Z., Liu L. (2017). Cancer cell membrane-biomimetic oxygen nanocarrier for breaking hypoxia-induced chemoresistance. *Advanced Functional Materials*.

[19] Xie W., Deng W. W., Zan M. (2019). Cancer cell membrane camouflaged nanoparticles to realize starvation therapy together with checkpoint blockades for enhancing cancer therapy. *ACS Nano*.

[20] Cheng J., Zhu Y., Xing X. (2021). Manganese-deposited iron oxide promotes tumor-responsive ferroptosis that synergizes the apoptosis of cisplatin. *Theranostics*.

[21] Rocca J. D., Werner M. E., Kramer S. A. (2015). Polysilsesquioxane nanoparticles for triggered release of cisplatin and effective cancer chemoradiotherapy. *Nanomedicine*.

[22] Yang K., Yu G., Yang Z. (2021). Supramolecular polymerization-induced nanoassemblies for self-augmented cascade chemotherapy and chemodynamic therapy of tumor. *Angewandte Chemie (International Ed. in English)*.

[23] Xiang H., You C., Liu W., Wang D., Chen Y., Dong C. (2021). Chemotherapy-enabled/augmented cascade catalytic tumor-oxidative nanotherapy. *Biomaterials*.

[24] Chen X., Yin X., Zhan L. (2022). Organelle-specific anchored delivery system stretching a reversal of tumor hypoxia microenvironment to a combinational chemo-photothermal therapy. *Advanced Functional Materials*.

[25] Jiao L., Yan H., Wu Y. (2020). When nanozymes meet single-atom catalysis. *Angewandte Chemie, International Edition*.

[26] Zhu D., Zheng Z., Luo G. (2021). Single injection and multiple treatments: an injectable nanozyme hydrogel as AIEgen reservoir and release controller for efficient tumor therapy. *Nano Today*.

[27] Liu X., Zhong X., Li C. (2021). Challenges in cell membrane-camouflaged drug delivery systems: development strategies and future prospects. *Chinese Chemical Letters*.

[28] Ding K., Zheng C., Sun L., Liu X., Yin Y., Wang L. (2020). NIR light-induced tumor phototherapy using ICG delivery system based on platelet-membrane-camouflaged hollow bismuth selenide nanoparticles. *Chinese Chemical Letters*.

[29] Pan Z., Yang L., Jca C., Wxa C., Gao L., Jda C. (2020). Targeted pH-responsive polyion complex micelle for controlled intracellular drug delivery. *Chinese Chemical Letters*.

[30] Ye H., Wang K., Wang M. (2019). Bioinspired nanoplatelets for chemo-photothermal therapy of breast cancer metastasis inhibition. *Biomaterials*.

[31] Zhu D., Lyu M., Jiang W., Suo M., Huang Q., Li K. (2020). A biomimetic nanozyme/camptothecin hybrid system for synergistically enhanced radiotherapy. *Journal of Materials Chemistry B*.

[32] Duo Y., Liu Q., Zhu D. (2022). Proof of concept for dual anticancer effects by a novel nanomaterial-mediated cancer cell killing and nano-radiosensitization. *Chemical Engineering Journal*.

[33] Zhu D. M., Xie W., Xiao Y. S. (2018). Erythrocyte membrane-coated gold nanocages for targeted photothermal and chemical cancer therapy. *Nanotechnology*.

[34] Fang R. H., Kroll A. V., Gao W., Zhang L. (2018). Cell membrane coating nanotechnology. *Advanced Materials*.

[35] Fang R. H., Hu C. M., Luk B. T. (2014). Cancer cell membrane-coated nanoparticles for anticancer vaccination and drug delivery. *Nano Letters*.

[36] Chen M., Wang P., Jiang D., Bao Z., Quan H. (2021). Platelet membranes coated gold nanocages for tumor targeted drug delivery and amplificated low-dose radiotherapy. *Frontiers in Oncology*.

[37] Fu L. H., Hu Y. R., Qi C. (2019). Biodegradable manganese-doped calcium phosphate nanotheranostics for traceable cascade reaction-enhanced anti-tumor therapy. *ACS Nano*.

[38] Zhu D., Chen H., Huang C. (2022). H_2_O_2_ self-producing single-atom nanozyme hydrogels as light-controlled oxidative stress amplifier for enhanced synergistic therapy by transforming “cold” tumors. *Advanced Functional Materials*.

[39] Yang X., Yang Y., Gao F., Wei J.-J., Qian C.-G., Sun M.-J. (2019). Biomimetic hybrid nanozymes with self-supplied H+ and accelerated O_2_ generation for enhanced starvation and photodynamic therapy against hypoxic tumors. *Nano Letters*.

[40] Hao Y. N., Gao Y. R., Li Y., Fei T., Shu Y., Wan J. H. (2021). Ultrasmall copper–gallic acid nanodots for chemodynamic therapy. *Advanced Materials Interfaces*.

[41] Koo S., Park O. K., Kim J. (2022). Enhanced chemodynamic therapy by Cu-Fe peroxide nanoparticles: tumor microenvironment-mediated synergistic Fenton reaction. *ACS Nano*.

