# Supplementary material for: Cancer Cell Membrane Biomimetic Mesoporous Nanozyme System with Efficient ROS Generation for Antitumor Chemoresistance
Source: Oxid Med Cell Longev. 2022 Oct 7;2022:5089857. doi: 10.1155/2022/5089857 (PMC9568328; doi:10.1155/2022/5089857)
Supplement: Supplementary Materials — Figure S1: EDX of Fe SAZs. Figure S2: cancer cell membrane marker, Epcam, was detected using western blotting. Figure S3: the activity analysis of NADPH oxidase (NOX) in HGC27/DDP cells after treated with 10 μg/mL DDP for different time points. Figure S4: nanoparticle uptake by HGC27/DDP cells at different concentrations. [file 5089857.f1.docx]

**Experimental Procedures**

**Materials and reagents**

Pluronic F127 (Mav = 12600), dopamine hydrochloride, FeCl_3_·6H_2_O and 3, 3’, 5, 5’- Tetramethylbenzidine (TMB) were from Sigma-Aldrich. Ammonium hydroxide (NH_4_OH, 28-30 wt%) and ethanol (C_2_H_6_O) were from Shanghai Chemical Corp. 3’-(4-Hydroxyphenyl) fluorescein (HPF) was purchased from Sigma–Aldrich (China). Cisplatin (DDP), Mitochondrial Membrane Potential Assay Kit with JC-1, Dopamine hydrochloride, DiO (DiOC18(3)) and Dil (DiIC18(3)) were obtained from Solarbio Life Science. All of the aqueous solutions were prepared using purified deionized (DI) water purified with a purification system (Direct-Q3, Millipore, USA). The other solvents used in this work were purchased from Sinopharm Chemical Reagent (China) and Aladdin-Reagent (China).

**Cell culture**

The HGC27 human gastric cancer cell line was obtained from the Cell Bank of the Chinese Academy of Sciences and incubated in RPMI-1640 medium supplemented with 10% FBS in a humidified atmosphere at 37 °C.

**Cisplatin-Resistant Cell Construction(**[**1**](#_ENREF_1)**)**

Gastric cancer cell line (HGC27) was seeded into a 6-well plate and cultured in fresh medium containing cisplatin at an initial concentration of 0.2 μg/mL. Then, observed the cells growth condition and cultured in cisplatin at the previous concentration until the HGC27 can grow steadily. Subsequently, increase the dose of cisplatin and repeat the procedure until cells could grow well in the presence of 1 μg/mL, then we obtained the cisplatin-resistant gastric cancer cell line (HGC27/DDP) that were tolerant to 1 μg/mL cisplatin.

**Synthesis of mesoporous carbon sphere precursors**

The synthesis of mesoporous carbon sphere precursors was achieved by a versatile nanoemulsion assembly approach 30. Specifically, 1.0 g of F127 and 0.5 g of DA were

added in a 100 mL mixture of water and ethanol (volume ratio 1:1). After stirring to a clear solution at room temperature, 0.7 mL 1, 3, 5-trimethylbenzene was gently dropped

into the solution and the stirring speed was then kept to 500 rpm. After stirring for 30 minutes, 2.0 mL concentrated ammonia (NH_4_OH) was then added and the reaction system was kept stirring for another 2 hours. The precursors were obtained by centrifuging and washing with ethanol for at least eight times, following freeze-drying for at least 12 hours.

**Synthesis of Fe SAZs**

200 mg precursors were dispersed in 15 mL water under ultrasound for at least 5 minutes. Then 600 μL FeCl_3_.6H_2_O aqueous solution (the concentration of Fe^3+^ is 10 mg/mL) was injected into the mixture under stirring. The afterward solution was then kept stirring for 12 h at room temperature. Products in this step were collected by centrifuging and 12-hour freeze-drying. The Fe-SAzyme was obtain by heating these powders at 600℃ for 4 hours (5 °C/min) under Ar atmosphere, with following sulphuric acid (0.5M) treatment for 6 hours.

**Characterization of Fe** **SAZs**

XRD was measured on a D8 Advance (Bruker- AXS, Germany). Energy dispersive X-ray spectroscopy (EDX) and scanning electron microscopy (SEM) of Fe SAZs were measured using a Hitachi S4800. Transmission electron microscopy (TEM) was recorded using an FEI Tecnai G2 S-Twin with a field emission gun operating at 200 kV. High-angle annular dark-field scanning transmission electron microscopy (HAADF-STEM) images and corresponding energy-dispersive spectroscopy (EDS) mapping analyses were acquired with a field-emission TEM (JEM-F200, Japan). The Fe single atom content was measured by ICP–AES.

**Peroxidase (POD)-like Activity of Fe SAZs**

Fe SAZs **(**0.1 mg/mL) were added to 84 μL of PBS (pH 7.4 or 6.5 or 5.5) containing 1 μL of TMB (20 mg/mL in DMSO) and 5 μL of H_2_O_2_ (10 mM). The UV−vis absorbance spectra of oxidized TMB were recorded. Then, Fe SAZs **(**0.1 mg/mL) were added to 84 μL of H_2_O_2_ (1mM or 2mM or 5mM) containing 1 μL of TMB (20 mg/mL in DMSO) and 85 μL PBS. The UV−vis absorbance spectra of oxidized TMB were recorded.

**Preparation of cancer cell membrane vesicles (CV)**

To prepare cancer cell membranes, we used a culture dish with 10 cm in diameter to culture HGC27/DDP cells at 37 °C, 5% CO_2_ and then used a cell scraper to detach the cells. Next, isolating the cells through a centrifugation treated at 720g for 6 min. Resuspending the collected cells into precooled PBS buffer (pH = 7.4) and centrifuging again at 600 g for 6 min. The collected cells pellets were resuspended in a hypotonic lysing buffer, including membrane protein extraction reagent and phenylmethanesulfonyl fluoride (PMSF) (Beyotine Institute of Biotechnology). Then, the sample above was incubated in ice bath for 10−15 min. Next, we used a freeze−thaw method repeatedly to break the cells in the above solution and then make a centrifugation at 700g for 10 min at 4 °C. To collect the cell membrane fragments, the

supernatant was subjected to further centrifugation at 14000g for 30 min. Subsequently, we used an Avanti mini extruder to extrude 11 times. Finally, we centrifuge to remove the redundant CV.

**Preparation and characterization of the biomimetic single-atom nanozyme system (CSD)**

Briefly, CV derived from HGC27/DDP cells was first mixed with 100 μg of Fe SAZs and 50 μg DDP in 1 mL of PBS, after which the mixture was extruded through a 200 nm membrane in a mini-extruder. The resultant particles were then spun for 10 min at 1,000 × g to remove the remaining CV, and the resultant CSD were collected and stored at 4 °C in PBS for subsequent experiments. A similar approach was used to synthesize a red blood cell membrane-coated single-atom nanozyme system (RSD). RBC membranes were obtained via a previously reported low-osmosis method([2](#_ENREF_2)). All subsequent steps were identical to those used to synthesize CSD. CV-coated SAZs (CS) were also prepared in the same way, except that DDP was removed. The amount of DDP loaded into CSD was calculated from UV–vis spectra. The drug loading efficiency (DLE) was measured by the following formula: DLE = (weight of loading drug/weight of feeding drugs) ×100%([2](#_ENREF_2)). The size distribution and zeta potential of different formulations were measured by dynamic light scattering. The morphological structures of different formulations were observed by TEM (JEOL-2100). UV–vis spectra of different samples were recorded by the UV–vis spectrophotometry Lambda 35 (Perkin-Elmer). Peroxidase (POD)-like activity of CSD was measured as mentioned above. Fe SAZs and DDP was coated with cancer cell membrane extracted from HGC27 cell line to form CSD (HGC27).

**NADPH oxidase activity**

The activity of NADPH oxidase was determined in membrane fractions (50 μg of protein) incubated with 1 mM EGTA and 5 μM lucigenin in phosphate buffer, pH 7.0. The assay was initiated by the addition of 50 μM NADPH to the incubation mixture. Samples were counted immediately using a tabletop luminometer with sampling time every 6 s. Samples were counted over a period of 5 min, and the fluorescence values were recorded for over 2 min of stable readings and averaged for that sample

**DDP release study**

The in vitro DDP release profile from CSD was determined. One milliliter of CSD containing 5μg/mL DDP was added to the culture dish. To investigate the stimuli effect of H_2_O_2_ on the release behavior, the release experiment of CSD was initially performed with or without 5 μL of H_2_O_2_ (10 mM). At the appropriate time point, 100 μL of different samples was collected, and a UV–vis spectrophotometer was used to monitor the released DDP content.

***In vitro* cancer targeting study**

HGC27/DDP cells were seeded in 24-well plates and cultured for 12 h. Then, 100 μL of RSD, CSD(HGC27) or CSD (containing 5μg/mL DDP) was added to the medium. Then, the cells were incubated for 2 h at 37 °C and 5% CO_2_ and washed with PBS three times. The cells were then harvested, stained with DAPI and imaged by using a fluorescence microscope (IX81, Olympus, Japan). For different concentration of DDP in vitro cell uptake, the nanoparticles uptake was measured by ICP-AES as described above.

**•OH detection *in vitro***

HGC27/DDP cells (8 × 10^4^ per plate) were incubated with 5 different groups: (1) PBS; (2) CS; (3) DDP; (4) RSD; and (5) CSD. The DDP concentration was 5μg/mL in groups 3, 4 and 5. Then, the fluorescent dye HPF (10 μM) was added and co-incubated for 10 min at 37 °C. The cells were detected by a fluorescence microscope (IX81, Olympus, Japan).

**Mitochondria Membrane Potential (MMP) Characterization**

HGC27/DDP cells were cultured with 5 different groups: (1) PBS; (2) CS; (3) DDP; (4) RSD; and (5) CSD for 10 h followed by the replacement of the culture medium with serum-free medium containing 10 μM JC-1 and then incubated for another 30 min in the dark. The DDP concentration was 5μg/mL in groups 3, 4 and 5. Next, the culture medium was removed, and the cells were washed three times with PBS. The fluorescence of JC-1 (red fluorescence, Ex: 543 nm, Em: 650 nm; green, Ex: 488 nm, Em: 515 nm) were observed by CLSM.

**Intracellular ATP detection**

HGC27/DDP cells (8 × 10^6^ per plate) were incubated with four different groups: (1) PBS; (2) CS; (3) DDP; (4) RSD; and (5) CSD. The DDP concentration was 5μg/mL in groups 3, 4 and 5. After 12 hours of incubation, the supernatant was discarded, and the intracellular ATP content was measured by the enhanced ATP Assay Kit according to the instructions.

***In vitro* anticancer effect of CSD**

The anticancer effect was measured by MTT assay. HGC27/DDP cells (8 × 10^4^ per plate) were incubated with 3 different groups: (1) PBS; (2) DDP; (3) CS; (4) RSD; and (5) CSD. The DDP concentration was 5μg/mL in groups 2, 4 and 5. At the end of the incubation, 5 mg/mL MTT PBS solution was added, and the plate was incubated for another 4 h. Finally, the absorbance values of the cells were determined by using a microplate reader (Emax Precision, USA) at 570 nm. The background absorbance of the well plate was measured and subtracted. The cytotoxicity was calculated by dividing the optical density (OD) values of the treated groups (T) by the OD values of the control (C) (T/C × 100%)([3](#_ENREF_3), [4](#_ENREF_4)). The anticancer effect of CSD at different DDP concentration was measured by the same method.

**Construction of mouse tumor models**

Female Balb/c nude mice aged 4-5 weeks were purchased from Vital River Company (Beijing, China). A total of 100 μL of HGC27/DDP cell suspension (5×10^6^ cells) was subcutaneously injected into each mouse to establish the tumor models. The animal experiments were carried out according to the protocol approved by the Ministry of Health in People’s Republic of PR China and were approved by the Administrative Committee on Animal Research of the Shenzhen People's Hospital.

***In Vivo* Pharmacokinetics and Distribution Study**

The HGC27/DDP tumor model was used. When tumors reached 400 mm^2^, the mice with subcutaneous tumors were i.v. injected with RPS (5 mg/kg DDP) or CSD (5 mg/kg DDP). Mice were sacrificed at 12 h after injection to collect the tumors and major organs for and weighed, the Pt content in the samples was analyzed using ICP–AES. For in vivo pharmacokinetics study. When tumors reached 400 mm^2^, the mice with subcutaneous tumors were i.v. injected with Fe SAZs or CSD (20 mg/kg Fe). At various time points after the injection (i.e.,0.5, 1, 2, 4, 8, 16, and 24 h), 20 μL blood was collected from the tail veins, the Fe content in the samples was analyzed using ICP–AES.

**Evaluation of intratumoral oxidative stress**

The HGC27/DDP tumor model was used. The mice were first divided randomly into 4 groups (each group included 3 mice): (1) PBS; (2) CS; (3) RSD; and (4) CSD. The DDP dose was 5 mg/kg in groups 3 and 4. HPF (10μg/mL, 50μL) was injected intratumorally 12 h after intravenous injection. Subsequently, tumors from each group were dissected. The cryosections were stained with HPF and observed by a confocal laser scanning microscope (CLSM; IX81, Olympus, Japan).

***In vivo* antitumor study**

The mice were first divided randomly into 4 groups (each group included 3 mice): (1) PBS; (2) CS; (3) RSD; and (4) CSD. The DDP dose was 5 mg/kg in groups 4 and 5. The treatment was conducted every 2 days for 16 days (8 treatments in total). After 16 days of treatment, all the mice were sacrificed. Five main organs (heart, liver, spleen, lung and kidney) and tumors of all mice were harvested, washed with PBS, and fixed with 4% paraformaldehyde for histological analysis. The tumor tissues were weighed, fixed in 4% neutral buffered formalin, processed routinely into paraffin, and sectioned at 4 μm. Then, the sections were stained with terminal deoxynucleotidyl transferase-mediated deoxyuridine triphosphate nick end labeling (TUNEL) and Ki-67 and finally examined by using a confocal laser scanning microscope (CLSM; IX81, Olympus, Japan).

**Statistical analysis**

Experimental data were analyzed by using one-way ANOVA followed by the post-Tukey comparison tests with GraphPad Prism 5.0 software. P < 0.05 indicates statistical

difference. *P < 0.05, **P < 0.01, ***P < 0.005.

**Supplementary figures**


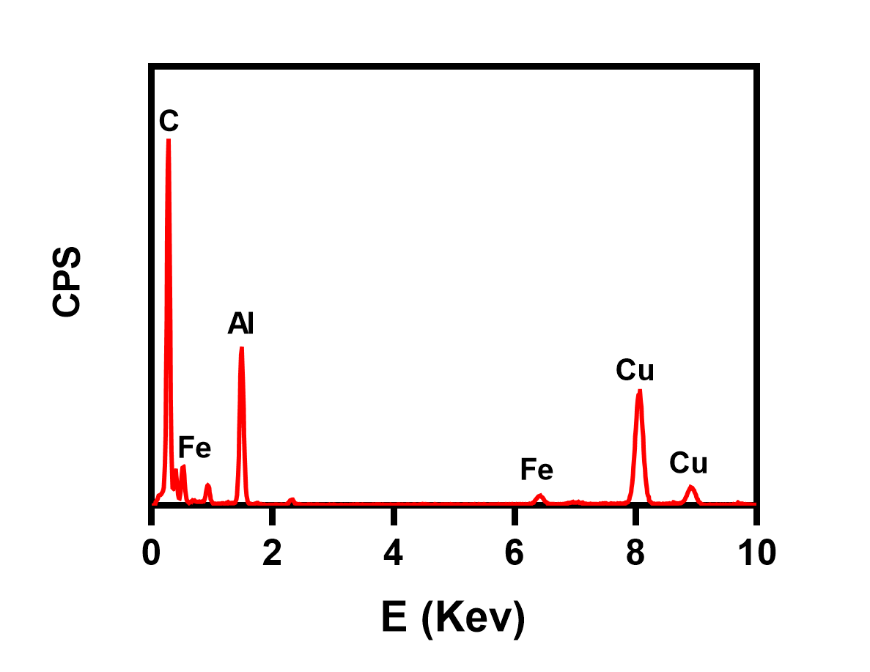


**Figure S1.** EDX of Fe SAZs.


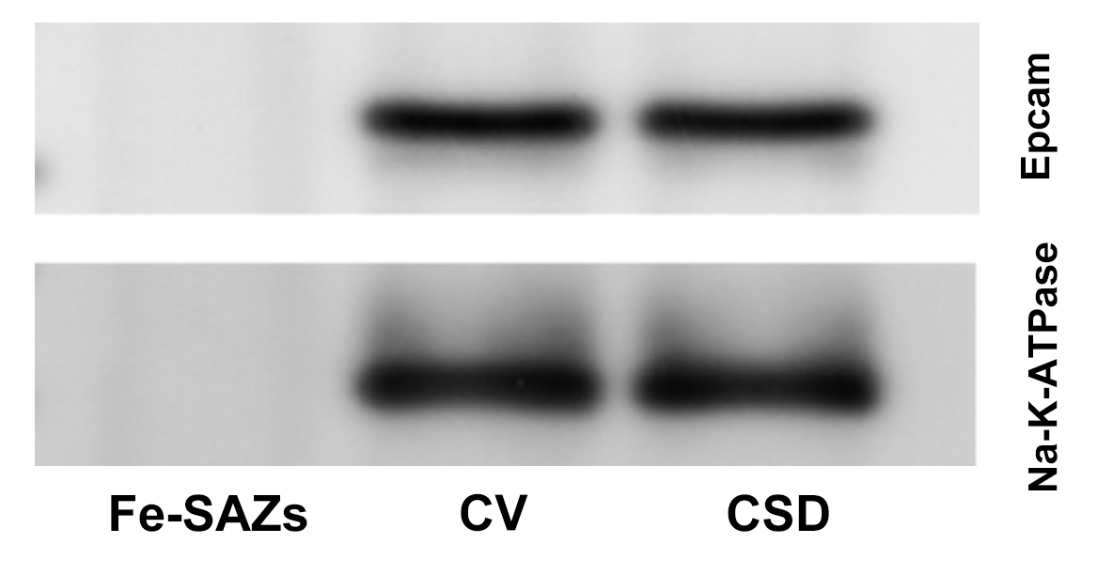


**Figure S2.** Cancer cell membrane marker, Epcam, was detected using western blotting.





**Figure S3.** The activity analysis of NADPH oxidase (NOX) in HGC27/DDP Cells after treated with 10 μg/mL DDP for different time points.





**Figure S4.** Nanoparticles uptake by HGC27/DDP cells at different concentration.

**References**

1. Y. Liu, H. Lei, J. Ma, H. Deng, P. He and W. Dong: alpha-Hederin Increases The Apoptosis Of Cisplatin-Resistant Gastric Cancer Cells By Activating Mitochondrial Pathway In Vivo And Vitro. *Onco Targets Ther*, 12, 8737-8750 (2019) doi:10.2147/OTT.S221005

2. D. M. Zhu, W. Xie, Y. S. Xiao, M. Suo, M. H. Zan, Q. Q. Liao, X. J. Hu, L. B. Chen, B. Chen, W. T. Wu, L. W. Ji, H. M. Huang, S. S. Guo, X. Z. Zhao, Q. Y. Liu and W. Liu: Erythrocyte membrane-coated gold nanocages for targeted photothermal and chemical cancer therapy. *Nanotechnology*, 29(8), 084002 (2018) doi:10.1088/1361-6528/aa9ca1

3. D. Zhu, Z. Zheng, G. Luo, M. Suo, X. Li, Y. Duo and B. Z. Tang: Single injection and multiple treatments: An injectable nanozyme hydrogel as AIEgen reservoir and release controller for efficient tumor therapy. *Nano Today*, 37, 101091 (2021) doi:10.1016/j.nantod.2021.101091

4. D. Zhu, Y. Duo, S. Meng, Y. Zhao, L. Xia, Z. Zheng, Y. Li and B. Z. Tang: Tumor-Exocytosed Exosome/Aggregation-Induced Emission Luminogen Hybrid Nanovesicles Facilitate Efficient Tumor Penetration and Photodynamic Therapy. *Angew. Chem., Int. Ed.*, 59, 2-10 (2020) doi:10.1002/anie.202003672

10.1002/ange.202003672
